# The Survival of the Kiss: Presence and Persistence of Salivary Male DNA in Mixed Samples

**DOI:** 10.3390/genes16020232

**Published:** 2025-02-19

**Authors:** Mauro Pesaresi, Federica Alessandrini, Elena Bignozzi, Alessia Bernini Di Michele, Filomena Melchionda, Rosaria Gesuita, Valerio Onofri, Chiara Turchi

**Affiliations:** 1 Section of Legal Medicine, Department of Biomedical Sciences and Public Health, Marche Polytechnic University, 60126 Ancona, Italy; m.pesaresi@univpm.it (M.P.); f.alessandrini@univpm.it (F.A.); e.bignozzi@staff.univpm.it (E.B.); a.bernini@pm.univpm.it (A.B.D.M.); f.melchionda@staff.univpm.it (F.M.); 2 Center of Epidemiology, Biostatistics and Medical Information Technology, Marche Polytechnic University, 60126 Ancona, Italy; r.gesuita@staff.univpm.it; 3 IRCCS INRCA, 60127 Ancona, Italy; 4 Legal Medicine Unit, Azienda Ospedaliero-Universitaria delle Marche, 60126 Ancona, Italy; valerio.onofri@ospedaliriuniti.marche.it

**Keywords:** saliva, kissing, DNA persistence, mixed profiles, qPCR, autosomal STR, Y-STR

## Abstract

Background/Objectives: The study of DNA transfer and persistence has become increasingly significant, driven by advancements in DNA detection sensitivity and the need for reliable forensic evidence. In forensic investigations, saliva and saliva-stained materials are recognised as valuable DNA sources, particularly in cases of homicide, sexual assault, and burglary, where saliva can be transferred between individuals during the criminal act. The time between the crime and sample collection is a critical factor that can influence the success of the analysis. The value of the specimens collected from the victim’s skin or mouth (perilabial and labial sites, teeth and tongue) after the crime has not been investigated with currently used highly sensitive and specific molecular methods. Methods: On the assumption that a significant loss of DNA occurred, in our study, 10 voluntary pairs were tested at different time points after intense kissing and samples were taken from the above-mentioned sites to assess the presence of the donor’s DNA. Extracted DNA was quantified using the Plexor HY System kit (Promega), and both autosomal STRs and Y-STRs were analysed. Results: The results reveal a greater persistence of male DNA on the female partner, particularly in the labial and perilabial regions, even up to 120 min after contact, in terms of both concentration and duration. Conclusions: This study emphasises the forensic importance of salivary DNA as a solid source of evidence, particularly in investigations involving mixed DNA profiles.

## 1. Introduction

Studying DNA transfer and the persistence of exogenous DNA has become increasingly important, due to the increasing sensitivity of DNA detection methods and to ensure that sound expert opinion evidence is provided. During sexual assaults, the perpetrator could constrain the victim to kissing, resulting in the transfer of DNA, which could be used both for personal identification and to establish the time when the fact happened.

Our study arises from a casework involving a man suspected of having killed his wife. The man claimed that the woman had suddenly disappeared after having a coffee together, and 60 h after her disappearance, her body was found in a forest. During the autopsy, a set of swabs was collected from the perilabial region and inside the buccal vestibule (teeth and tongue). DNA quantification performed by the real-time PCR assay Plexor^®^ HY System (Promega, Madison, WI, USA) displays an autosomal DNA concentration (ng/μL) of 0.545, 3.763, and 3.933 and Y chromosome DNA concentration (ng/μL) of 0.393, 0.235, and 0.025 in the perilabial region, teeth, and tongue swabs, respectively. Autosomal short tandem repeat (STR) typing performed on perilabial region and teeth swabs resulted in mixed-DNA profiles, while Y-STR typing resulted in a single male profile matching with the man’s profile.

Saliva is a biological fluid consisting of 99% water, and the remaining 1% contains an entire library of electrolytes, hormones, proteins, enzymes, antibodies, antimicrobial constituents, and cytokines, with an osmolarity similar to that of plasma [1]. The greatest part of saliva is produced by the major salivary glands (parotid, submaxillary, and sublingual), while a small contribution is given by the minor salivary glands (labial, buccal, and palatine) [1]. The average daily flow of whole saliva varies in health between 1 and 1.5 L. Percentage contributions of the different salivary glands during unstimulated flow are as follows: 20% from parotid, 65% from submandibular, 7% to 8% from sublingual, and less than 10% from numerous minor glands. Stimulated high flow rates drastically change percentage contributions from each gland, with the parotid contributing more than 50% of total salivary secretions [2]. DNA is not present in the liquid saliva but in the cellular material, such as epithelial cells and glandular cells, which are naturally sloughed from the inner lining of the mouth [3].

In forensic science, saliva is a type of trace evidence frequently encountered at crime scenes, characterised by its organic and inorganic components, which make it comparable to blood. Due to its ease of collection, the non-invasive nature of its sampling, and the relatively low cost of analysis, saliva represents a viable alternative to blood in forensic investigations, offering an effective means for individual identification and the reconstruction of criminal events [4,5].

During a criminal act, there may be a transfer of genetic material from the victim to the perpetrator and vice versa. In cases of sexual violence, sexual abuse, or sexual harassment, a transfer of salivary DNA may occur as a result of kissing, licking, spitting, biting, or sucking [6,7]. For this reason, saliva and saliva-stained materials can be a good source of evidence in police investigations as they can provide important information to forensic scientists useful for the identification of the perpetrator through DNA profiling [3,7].

The persistence of transferred material is constantly a question of relevance to forensic scientists [8]. Persistence studies can be used to help scientists assess the expected level of a given type of trace evidence remaining after a certain time. A study carried out by Sweet et al. [6] demonstrated the persistence of saliva on cadaver tissue and the ability to recover DNA for up to 48 h following application. Although a significant loss of DNA occurred between recovery after 5 min and recovery after 24 h, very little loss occurred between 24 and 48 h, demonstrating the robust nature of DNA and the stability of this molecule following dehydration of saliva. The initial loss may be attributed to degradation of the DNA or inefficient recovery due to dehydration of the nucleated cells on the tissue of the corpse. Banaschak et al. [9] reported the detection of male DNA in female samples only up to 60 s after the exchange of a kiss.

Anzai-Kanto et al. [10] published a study of 20 saliva samples collected from different donors and used as suspects’ samples. Five of these samples were randomly selected and deposited (250 μL) on arm skin. The recovery of DNA from saliva deposited on the skin was 10- to 14-times lower than the DNA quantity from saliva samples. DNA typing was demonstrated in four of five deposited saliva samples. In this study, the transfer of saliva from one individual to the skin of another individual was simulated by depositing previously spitted saliva on the skin. In real cases, saliva is deposited by direct contact between the mouth of an individual and the skin of the other; in this way, numerous buccal cells can be transferred to the skin, thus increasing the amount of DNA present and recoverable.

The study of Kenna et al. [3], where saliva from three male donors was deposited on the skin of three female recipients, demonstrated that a full male DNA profile was obtained even after 96 h. The amount of male salivary DNA remaining on the female skin was measured over a 96 h period, and in eight of the nine experiments, a full male DNA profile matching the donor was obtained even after 96 h. In addition, the study showed that the concentration of salivary DNA varied from donor to donor and from day to day.

A subsequent study conducted by Kamodyová et al. [7] demonstrated that male exogenous DNA could persist for up to 60 min post-kiss. Furthermore, a complete Y-STR genotype was successfully obtained from samples collected as early as 10 min after the kiss.

Several techniques have been used in the past to find the best method of recovering saliva from human skin. A recent study [11] compared the single- and double-swab techniques and founded no significant difference between the two methods.

Correct DNA quantification is an essential aspect of obtaining reliable results in subsequent genotyping analysis, and various commercial kits are used to obtain a quantitative and qualitative assessment of total human DNA in a single, highly sensitive real-time PCR reaction [12].

STR profiling represents a highly effective forensic technique used for individual identification and for mixed-DNA trace analysis [13]. This feature is of particular importance in cases of sexual assault, where the likelihood of obtaining mixed profiles from traces found at the crime scene is extremely high. When in a mixed profile the female DNA is quantitatively greater than the male DNA, Y-STR marker profiling in combination with autosomal STR analysis is effective for the detection and quantification of the male component. The quantification of male Y-DNA is a rapid and effective method for assessing the persistence of male DNA, without interference from female genetic material [3,7]. However, Y-STR analysis alone cannot discriminate between individuals of the same paternal lineage, even if the Y-STR profile found at the crime scene matches the profile of the suspect [14]. Despite this limitation, Y-STR haplotyping remains an invaluable tool in crime scene investigation as it not only helps in identifying potential male suspects but also aids in excluding innocent male individuals from suspicion [15]. Since the success of the analysis is affected by the time elapses between the commission of the criminal act and the collection of the sample, the utility of persistence studies lies in their capacity to estimate the quantity of DNA in a specific type of trace that remains after a designated time [7].

The aim of our study was to investigate the presence and persistence of male DNA in female saliva samples, and vice versa, using DNA quantification techniques via real-time PCR and the analysis of autosomal STRs and Y-chromosome STR markers. The research focuses on female:male DNA mixtures, obtained from samples collected in different oral and perioral regions following the exchange of a kiss. This may be of interest to forensic experts and criminalists in estimating the critical time for the detection of transferred DNA in a mixed-saliva sample following the contact between the victim and the perpetrator. Moreover, the results of this study could be useful in the evaluation of saliva traces considering activity-level propositions.

## 2. Materials and Methods

### 2.1. Sample Collection

Twenty volunteers participated in this study after being fully informed about its concept and providing signed informed consent. Ten pairs of healthy volunteers, each comprising a man and a woman, aged between 22 and 40 years, exchanged a French kiss for 5 s after a 24 h period of no contact between them, and two hours after brushing their teeth, to ensure that the recovered material was derived from the exchange of biological material through the kiss. Males and females were instructed not to eat, drink, shower, or wash the areas where saliva was going to be collected: teeth, tongue, lips, and perilabial area.

To test the persistence of the exchanged DNA on the skin, saliva was collected from the oral cavity of the subjects using a swab technique on 7 different days, each day at a different time interval. The subjects kissed each time, following the recommendations for the test, and saliva samples were collected immediately after the kiss (t0) and 5 (t5), 10 (t10), 15 (t15), 30 (t30), 60 (t60), and 120 (t120) minutes after kissing. An oral swab was taken before the kiss to exclude any contamination. As couple 10 withdrew from the study prior to its conclusion, only data up to time point 15 were collected.

### 2.2. Sample Processing

DNA was extracted using the DNA IQ™ Casework Pro Kit for Maxwell^®^ 16 (Promega), according to the technical manual [16]. Subsequently, the purified samples were quantified using the Plexor HY System kit (Promega) [17] with the instrument Rotor-Gene™ Corbett 6000 (QIAGEN, Hilden, Germany) and analysed using Rotor-Gene™ 6000 software v.1.7. Samples were then amplified using the PowerPlex^®^ ESI 17 Pro System kit (Promega) [18] for autosomal DNA and AmpF*l*STR^®^ Yfiler^®^ PCR Amplification Kit (Applied Biosystems, Foster City, CA, USA) [19] for Y-chromosome DNA on a GeneAmp PCR System 9700 thermal cycler (Applied Biosystems, Foster City, CA, USA), as recommended by the manufacturer. Profiles were generated using an ABI PRISM 3130 Genetic Analyzer (Applied Biosystems). Analysis was performed using GeneMapper ID-X v.1.4 (Applied Biosystems). The analytical threshold and the stochastic threshold were set to 50 RFU (relative fluorescence units) and 200 RFU, respectively.

## 3. Results and Discussion

### 3.1. qPCR Results

**Figure 1:**
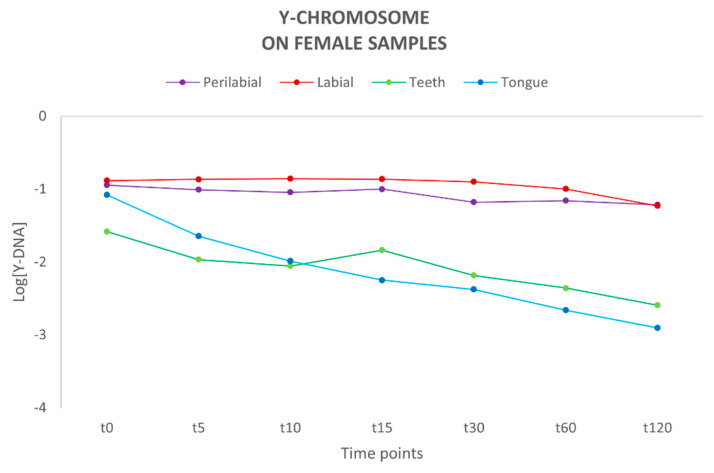
Amount of Y-chromosome DNA present on each female collection site (n = 10). Y-axis: values of Y-DNA are shown in a logarithmic scale (Log); x-axis: time points.

Following quantification using the Plexor HY System kit, the concentration of autosomal (total) DNA and Y-chromosome DNA was determined for each sample (Supplementary Tables S1 and S2). The autosomal DNA probe detects both male and female DNA, and thus it is not possible to distinguish the male component from the female one. Therefore, we focused on evaluating the quantity and persistence of Y-chromosome DNA on the female partner. Male DNA presence was observed up to 120 min after the kiss in all sites. The highest concentration of Y-DNA was detected at the labial and perilabial sites of the female partner, with a relatively constant trend over time, in contrast to teeth and tongue sites, where a decrease in genetic material was observed over time (Figure 1). A previous study by Kamodyová et al. [7] investigated the presence and persistence of DNA in mixed-saliva samples collected from the oral cavity following an intense kiss, detecting the presence of male DNA on female swabs up to 60 min post-contact.

**Figure 2:**
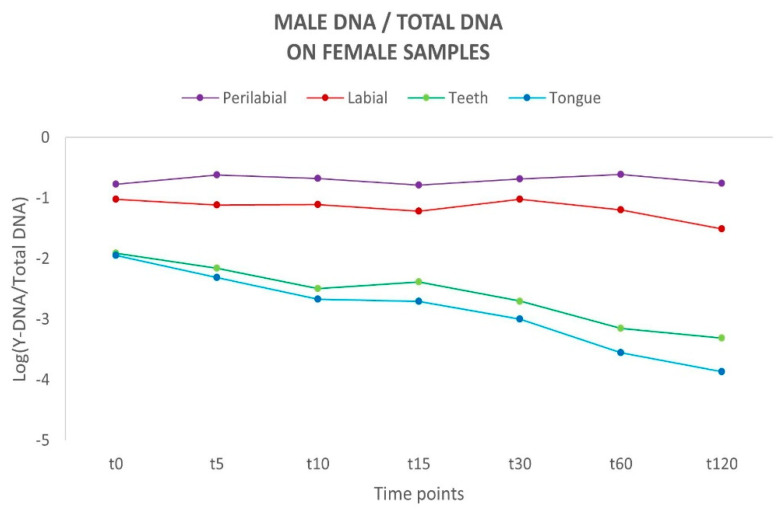
Schematic representation of the variation in male DNA recovered on female sites over time. Y-axis: values of Y-DNA/Total DNA ratio are shown in a logarithmic scale (Log); x-axis: time points.

To assess the proportion of male DNA and its persistence, the [Y/Auto] ratio was calculated and subsequently converted to a logarithmic scale (Log) to normalise the distribution and ensure uniformity. For all samples in all analysed sites, a negative Y/Auto ratio was observed, indicating that the amount of female DNA exceeded that of male DNA. Considering the different time intervals, a flat linear trend was observed for the labial and perilabial sites, suggesting a reduced loss of male genetic material. In contrast, a different trend emerged for the curves representing the teeth and tongue, which exhibit a tendency toward a rapid decline over time (Figure 2). Such differences between these areas align with the absence of a physiological removal process for biological material on the external sites of the oral cavity, unlike the active removal mechanisms observed in internal sites.

Among the couples, a marked difference in the quantity of male DNA recovered was observed, indicating a variable amount of male genetic material transferred during kissing. These data reflect an individual variability in the release of genetic material through kissing. Concerning the samples collected from the oral cavity (teeth and tongue), the differences between the couples may be attributed to a different ability to remove the partner’s genetic material due to differences in salivary flow and/or swallowing frequency among the female subjects. A common factor across all couples was the observation that DNA persistence was consistently higher at the perilabial and labial sites across all considered time intervals.

### 3.2. STR Results

Upon comparison with the reference profiles, discriminative alleles (i.e., alleles not shared between the autosomal profiles of samples within each couple) were identified. A minimum of 12 to a maximum of 19 discriminating alleles were found between samples within each couple. The presence and persistence of these discriminative alleles were then assessed across the sampling sites over the different time intervals (Supplementary Tables S3 and S4).

The amplification reaction confirmed the results of the quantification, as expected, showing the greatest amount of the partner’s DNA in the perilabial and labial sites; however, this tended to decrease over time.

**Figure 3:**
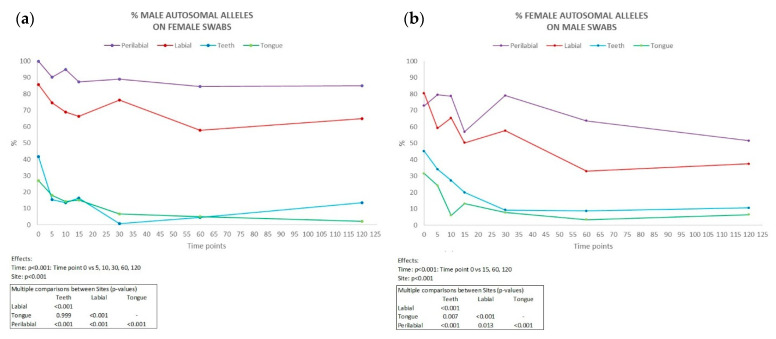
Percentage of male autosomal alleles detected on female partner (**a**) and female autosomal alleles detected on male partner (**b**) in all collection sites (n = 10) at each time interval considered (min). Results of ANOVA for repeated measures (ANOVA = analysis of variance). vs = versus.

Complete male autosomal profiles were obtained from the labial and perilabial female samples, even up to 120 min after kissing (in five couples in the perilabial site and two couples in the labial site), with an average percentage of detected alleles of 90.1% and 70.6%, respectively (Figure 3b). Conversely, in swabs collected from the male partner, the percentage of discriminative female alleles detected was lower (Figure 3a), with averages of 68.9% in the labial site and 54.8% in the perilabial site (complete genetic profiles in two couples in the perilabial site and one couple in the labial site 120 min after kissing). 

Inside the oral cavity, the number of the partner’s alleles was significantly lower compared to external sites, being completely absent in some couples. Across all couples, the persistence of male DNA on females was consistently higher than that of female DNA on males. This disparity was particularly pronounced in samples collected from perilabial sites. These findings are consistent with the existing literature, which suggests that female salivary flow is lower than that of males. This difference between males and females may be attributed to the fact that men tend to produce more saliva than women, possibly due to hormonal factors [20], the size of salivary glands, and body weight [21]. Our study would therefore confirm gender as a possible factor of inter-individual variability.

In some cases (couple 1), the male profile outnumbers that of the female partner depending on when the sample was taken (from t0 to t10), losing predominance at t15. In other couples, such as couple 2, the trend of the perilabial region is not constant but increases (t15). This could be explained either by a different release of partner DNA during the four kissing sessions or by a different emotional involvement between the pairs, although efforts were made to standardise the lip pressure.

In swabs taken from the teeth and tongue, discriminative alleles were detected in minimal quantities or were entirely absent in both the male and female samples, with an average percentage of 15.1% from the teeth and 12.6% from the tongue in female samples, and 22.2% from the teeth and 13.2% from the tongue in male samples.

**Figure 4:**
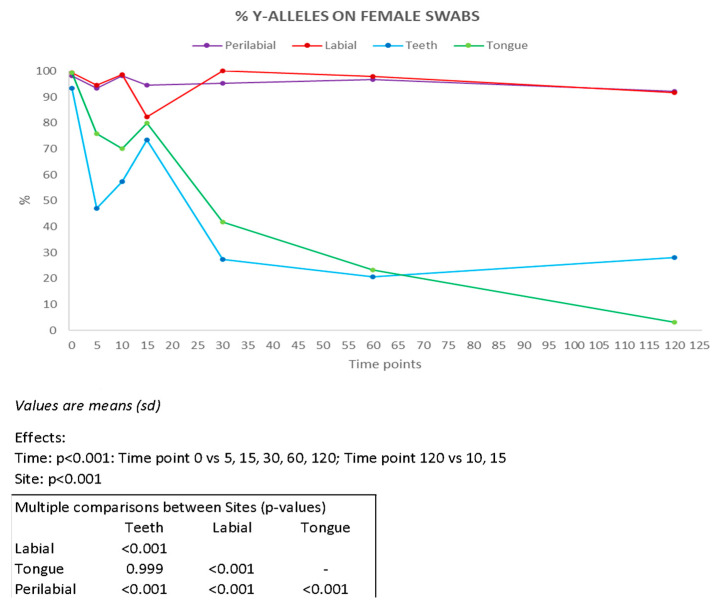
Percentage of Y-chromosome alleles recovered on female partners present on each collection site (n = 10) at all time intervals considered (min). Results of ANOVA for repeated measures (ANOVA = analysis of variance). vs = versus.

Y-STR profiles enabled the identification of the male component in female swabs (Figure 4). The most effective sampling sites were found to be the labial and perilabial areas, from which complete male profiles were obtained up to 120 min after the kiss, in six and eight samples, respectively (Supplementary Table S5). For samples collected from the teeth and tongue, complete male profiles were recovered up to 30 min after the kiss in one and two samples, respectively. Our results are consistent with those reported by Kamodyová et al. [7] who obtained complete male genetic profiles in four samples after 10 min and in one sample after 30 min.

## 4. Conclusions

This study underscores the forensic relevance of salivary DNA as a robust source of evidence for criminal investigations, particularly in cases involving close contact such as sexual assault. Significant differences were highlighted in the persistence of male DNA across various sampling sites, with a stronger permanence in the perilabial and labial sites even up to 120 min post-contact. These findings qualify these areas as optimal for forensic sampling, in contrast with the teeth and tongue, which exhibited a faster loss of male DNA, likely due to physiological active removal processes. The findings align with prior studies, extending the window of detectable male DNA and enhancing the understanding of DNA transfer dynamics. The differential persistence rates across sites may be influenced by biological and anatomical factors, including salivary flow and individual variation. These results provide valuable insights for refining forensic sampling strategies, particularly in time-sensitive investigations involving mixed-DNA profiles.

## Data Availability

The original contributions presented in this study are included in the article/Supplementary Materials. Further inquiries can be directed to the corresponding author.

## Supplementary Materials

The following supporting information can be downloaded at: https://www.mdpi.com/article/10.3390/genes16020232/s1, Supplementary Table S1: DNA concentration of autosomal (total) DNA and Y-chromosome DNA in FEMALE samples. Swabs were collected immediately after the kiss 0 (t0), 5 (t5), 10 (t10), 15 (t15), 30 (t30), 60 (t60), and 120 (t120) minutes after kissing. N/D = not defined; Supplementary Table S2: DNA concentration of autosomal (total) DNA and Y-chromosome DNA in MALE samples. Swabs were collected immediately after the kiss 0 (t0), 5 (t5), 10 (t10), 15 (t15), 30 (t30), 60 (t60), and 120 (t120) minutes after kissing. N/D = not defined; Supplementary Table S3: Percentage of MALE informative autosomal alleles detected on female oral swabs; Supplementary Table S4: Percentage of FEMALE informative autosomal alleles detected on male oral swabs; Supplementary Table S5: Percentage of Y-chromosome alleles detected on female oral swabs.

## References

[1] Humphrey S.P., Williamson R.T. (2001). A Review of Saliva: Normal Composition, Flow, and Function. J. Prosthet. Dent..

[2] Edgar W.M. (1990). Saliva and Dental Health. Clinical Implications of Saliva: Report of a Consensus Meeting. Br. Dent. J..

[3] Kenna J., Smyth M., McKenna L., Dockery C., McDermott S.D. (2010). The Recovery and Persistence of Salivary DNA on Human Skin. J. Forensic Sci..

[4] Upadhyay M., Shrivastava P., Verma K., Joshi B. (2023). Recent Advancements in Identification and Detection of Saliva as Forensic Evidence: A Review. Egypt. J. Forensic Sci..

[5] Kar A.K., Patel R., Debnath A. (2024). Introduction to Saliva and Its Forensic Analysis.

[6] Sweet D., Lorente J.A., Valenzuela A., Lorente M., Villanueva E. (1997). PCR-Based DNA Typing of Saliva Stains Recovered from Human Skin. J. Forensic Sci..

[7] Kamodyová N., Durdiaková J., Celec P., Sedláčková T., Repiská G., Sviežená B., Minárik G. (2013). Prevalence and Persistence of Male DNA Identified in Mixed Saliva Samples after Intense Kissing. Forensic Sci. Int. Genet..

[8] van Oorschot R.A.H., Szkuta B., Meakin G.E., Kokshoorn B., Goray M. (2019). DNA transfer in forensic science: A review. Forensic Sci. Int. Genet..

[9] Banaschak S., Möller K., Pfeiffer H. (1998). Potential DNA Mixtures Introduced through Kissing. Int. J. Leg. Med..

[10] Anzai-Kanto E., Hirata M.H., Hirata R.D., Nunes F.D., Melani R.F., Oliveira R.N. (2005). DNA extraction from human saliva deposited on skin and its use in forensic identification procedures. Braz. Oral Res..

[11] Graham E.A.M., Rutty G.N. (2008). Investigation into Normal Background DNA on Adult Necks: Implications for DNA Profiling of Manual Strangulation Victims. J. Forensic Sci..

[12] Seo S.B., Lee H.Y., Zhang A.H., Kim H.Y., Shin D.H., Lee S.D. (2011). Effects of Humic Acid on DNA Quantification with Quantifiler^®^ Human DNA Quantification Kit and Short Tandem Repeat Amplification Efficiency. Int. J. Leg. Med..

[13] Clayton T.M., Whitaker J.P., Sparkes R., Gill P. (1998). Analysis and Interpretation of Mixed Forensic Stains Using DNA STR Profiling. Forensic Sci. Int..

[14] Roewer L. (2009). Y Chromosome STR Typing in Crime Casework. Forensic Sci. Med. Pathol..

[15] Kayser M., Caglià A., Corach D., Fretwell N., Gehrig C., Graziosi G., Heidorn F., Herrmann S., Herzog B., Hidding M. (1997). Evaluation of Y-Chromosomal STRs: A Multicenter Study. Int. J. Leg. Med..

[16] Promega DNA IQ™ Casework Pro Kit for Maxwell^®^ 16; Technical Manual TM332, Revised 12/16. https://ita.promega.com/-/media/files/resources/protocols/technical-manuals/101/dna-iq-casework-pro-kit-for-maxwell-16-protocol.pdf?rev=fde31f63a4114dc5b9088112ab8cddeb&sc_lang=en.

[17] Promega Plexor^®^ HY System for Applied Biosystems 7500 and 7500 Fast Real-Time PCR Systems, Technical Manual TM293, Revised 9/17. https://ita.promega.com/-/media/files/resources/protocols/technical-manuals/0/plexor-hy-system-for-the-applied-biosystems-7500-and-7500-fast-real-time-pcr-systems-protocol.pdf?rev=ee860374bdb944e18c1e41ef075600ba&sc_lang=en.

[18] Promega PowerPlex^®^ ESI 17 Fast System for Use on the Applied Biosystems^®^ Genetic Analyzers; Technical Manual TMD041, Revised 2/21. https://ita.promega.com/en/products/forensic-dna-analysis-ce/str-amplification/powerplex-esx-17-and-esi-17-fast-systems/?catNum=DC1710.

[19] Applied Biosystems by Life Technologies AmpFlSTR Yfiler PCR Amplification; User Guide (Pub. no. 4358101). https://assets.thermofisher.com/TFS-Assets/LSG/manuals/4358101_AmpflstrYfilerKit_UG.pdf.

[20] Ekström J., Khosravani N., Castagnola M., Messana I. (2011). Saliva and the Control of Its Secretion. Medical Radiology.

[21] Inoue H., Ono K., Masuda W., Morimoto Y., Tanaka T., Yokota M., Inenaga K. (2006). Gender Difference in Unstimulated Whole Saliva Flow Rate and Salivary Gland Sizes. Arch. Oral Biol..

